# When it rains, it pours: detecting seasonal patterns in utilization of maternal healthcare in Mozambique using routine data

**DOI:** 10.1186/s12913-020-05807-0

**Published:** 2020-10-15

**Authors:** Briana Stone, Júlia Sambo, Talata Sawadogo-Lewis, Timothy Roberton

**Affiliations:** 1 Johns Hopkins Bloomberg School of Public Health, Baltimore, MD USA; 2 Instituto Nacional de Saúde, Ministério da Saúde, Maputo, Mozambique

**Keywords:** Seasonality, Mozambique, Maternal health, Health care access

## Abstract

**Background:**

Climatic conditions and seasonal trends can affect population health, but typically, we consider the effect of climate on the epidemiology of communicable diseases. However, climate can also have an effect on access to care, particularly in remote rural areas of low- and middle-income countries. In this study, we investigate associations between the rainy season and the utilization of maternal health services in Mozambique.

**Methods:**

We examined patterns in the number of women receiving antenatal care (ANC) and delivering at a health facility for 2012–2019, using data from Mozambique’s Health Management Information Systems. We investigated the association between seasonality (rainfall) and maternal health service utilization (ANC and institutional delivery) at national and provincial level. We fit a negative binomial regression model for institutional delivery and used it to estimate the yearly reduction in institutional deliveries due to the rainy season, with other factors held constant. We used the Lives Saved Tool (LiST) to model increases in mortality due to this estimated decrease in institutional delivery associated with the rainy season.

**Results:**

In our national analysis, the rate of ANC visits was 1% lower during the rainy season, adjusting for year and province (IRR = 0.99, 95% CI: 0.96–1.03). The rate of institutional deliveries was 6% lower during the rainy season than the dry season, after adjusting for time and province (IRR = 0.94, 95% CI: 0.92–0.96). In provincial analyses, all provinces except for Maputo-Cidade, Maputo-Province, Nampula, and Niassa showed a statistically significantly lower rate of institutional deliveries in the rainy season. None were statistically significantly lower for ANC. We estimate that, due to reductions in institutional delivery attributable only to the rainy season, there were 74 additional maternal deaths and 726 additional deaths of children under the age of 1 month in 2021, that would not have died if the mothers had instead delivered at a facility.

**Conclusion:**

Fewer women deliver at a health facility during the rainy season in Mozambique than during the dry season. Barriers to receiving care during pregnancy and childbirth must be addressed using a multisectoral approach, considering the impact of geographical inequities.

## Background

In countries with marked changes in climatic conditions depending on season, these conditions can determine epidemiologic disease patterns of “seasonal” diseases. For example, malaria or cholera incidence are expected to spike during the rainy season, due to increases in mosquito populations and stagnating waters caused by the high volume of rain [1–3]. However, seasonal pattern in disease incidence or health outcome is not limited to diseases with such a direct relationship to weather-related events. Indeed, existing studies have shown that fluctuations in access and utilization of health services are also linked to the ability to travel to a health facility at different times of the year [4–7].

Transportation and distance-related issues are commonly reported barriers to accessing health care services in Mozambique [8, 9]. According to Mozambique’s most recent Demographic and Health Survey (DHS 2011), the biggest problem of access to health care reported by women is the distance to health unit. For example, in Zambezia, 80% of women said distance was a problem for accessing health care [10]. In a study investigating healthcare service availability in Mozambique, an average of 40% of the population live 2 or more hours of walking distance from the closest health facility [11]. Difficulty accessing health care is exacerbated when rain floods the roads, making transportation more difficult and causing further delays [12]. A qualitative study in rural Mozambique described transportation and poor condition of roads as barriers to assessing health facilities in Zambezia, with one participant describing a full day’s journey of 150 km to access an antiretroviral therapy clinic the next day [13]. Roads in Mozambique are mostly unpaved and vulnerable to the floods and heavy precipitation frequently experienced in Mozambique, with large sections of the few paved roads virtually impassable during the rainy season [14]. The impact of severe weather on road infrastructure resulting in long travel times and, in some cases, isolation from health facilities has also been documented [15].

Maternal deaths are a result of complications during and following pregnancy and childbirth, and most are preventable through the provision of basic health services – including ensuring that every birth occurs with the assistance of skilled health personnel [16]. In 2002, Mozambique signed up to the millennium development goals (MDGs), which included a commitment to reduce the maternal mortality ratio by three quarters between 1990 and 2015. Mozambique committed to the Sustainable Development Goals (SDGs) in 2015, which includes ensuring healthy lives and promoting well-being for all ages, and implemented the 2030 Agenda, including all 17 SDGs, into the framework of Mozambique’s national development plan in 2017. Despite a 50% reduction since 1990 [17], maternal mortality remains at a concerningly high rate (489/100,000 live births) [18] .

Mozambique’s Health Sector Strategic Plan (PESS, Plano Estratégico do Sector da Saúde) for 2014–2019 included accelerating progress in the reduction of maternal and neonatal mortality as one of Mozambique’s health priorities [19]. To accelerate progress of access to sexual and reproductive health programs specifically, the PESS included strategies and interventions intended to increase demand for antenatal (ANC) and institutional deliveries. These include encouraging women through community involvement to attend at least 4 ANC visits during the course of their pregnancy, constructing “maternity waiting homes” at health facilities where women can stay while waiting for labor to start, and using Traditional Birth Attendants (TBA) and community health worker participation in referral systems for pregnant women and women during or after labor [19]. It is clear that in Mozambique, the government has made a commitment to the reduction of maternal mortality and prioritized improved demand for institutional deliveries.

A limited number of studies have analyzed how the rainy season in Mozambique or neighboring countries affects access to maternal health care services [5, 15, 20]. To the best of our knowledge, no study has specifically attempted to identify seasonal patterns in the delivery of maternal health services using routine data. Because it is collected on an ongoing, uninterrupted basis, routine data is especially beneficial for identifying and analyzing trends.

In this study, we sought to quantitatively investigate the effect of the rainy season on utilization of maternal health services at a health facility. We hope that these findings will help inform decision-makers as they decide on the allocation of resources to address bottlenecks in improving maternal health outcomes in Mozambique.

## Methods

### Data sources

We obtained routine monthly count data from the national health management information system (HMIS) for at least four completed ANC visits (ANC4) and count data of women delivering at health facilities (institutional delivery) in each of the 11 provinces, including the capital, Maputo-Cidade.

Data for January 2012 to December 2015 came from Mozambique’s HMIS, *Modulo Basico*. The HMIS transitioned to the DHIS-2-based system called *Sistema de Informação para a Saúde–Monitoria e Avaliação* (SIS-MA) in 2016, and data from January 2017 to August 2019 comes from SIS-MA. The first 5 months of 2012 were excluded to account for slow adoption of the HMIS system in Mozambique. Of note, there were delays in the implementation of the systems transition activities, and completeness of the data reported through the SIS-MA remains a concern [21]. We were not able to obtain data for the year of transition (2016).

Meteorological data was derived from the Climate Hazards Group InfraRed Precipitation with Stations (CHIRPS) dataset via the USAID Famine Early Warning Systems Network [22]. The publicly available CHIRPS dataset contains daily rainfall estimates from rain gauge and satellite observations for each province, excluding the capital Maputo-Cidade. Because weather station density over Mozambique is low and rainfall data collected is therefore very dependent on proxy satellite data for large areas, rainfall data was used to determine seasons.

### Covariates

Precipitation data was retrieved for the study period, including monthly rainfall, which ranged from 2.40 to 538.60 mm from January 2012 to August 2019. A binary seasonality predictor for rainy and dry season was created based on average rainfall per month (Additional File 1). We categorized the rainy season as January, February, March, and December, the months with the highest rainfall, and all other months as being the dry season. A binary predictor variable was created for SIS-MA after 2016 versus the older HMIS, *Modulo Basico,* to account for any changes due to the new system*.* Nampula, the province with the largest population, represented the reference category for provinces.

### Outcome variables

We assessed (1) frequency of ANC visits, calculated as the monthly total number of pregnant women completing 4 ANC visits at a health facility, and (2) the total number of pregnant women delivering at a health facility each month as outcome variables. We fit separate regression models for each outcome to assess whether or not being in the rainy season would decrease the frequency of maternal health service-related visits.

### Analysis

We conducted statistical analyses of the association between seasonality and counts of maternal health facility visits at the national and provincial level. We first looked at the distribution of completed ANC4 visits and institutional deliveries, including the frequency of zero counts, and examined summary statistics, such as mean, median, skewness, and variance. We checked for collinearity by investigating viariance inflation factors (VIF). As VIF for both models were well below 2.00, we assumed that collinearity to be negligible for the models fitted here.

To determine the most appropriate approach for examining the association between rainy season and number of facility visits, we evaluated a number of regression models, including the Poisson model, a negative binomial mean-dispersion model, and a generalized linear model (GLM) assuming an overdispersed Poisson model.

After evaluating the predictive performance of each model for each outcome using Akaike information criterion (AIC), we found that the negative binomial model showed the best model fit to the data. We fit the overall model for each outcome (counts of ANC; counts of institutional deliveries), adjusted for time (monthly), to account for unmeasured confounders that may vary over time, HMIS change, and region (as provinces). Accounting for heterogeneity across province in both frequency of facility visits and precipitation across Mozambique, provincial associations were estimated by stratifying by provinces in our model.

We conducted all analyses using Stata version 15.1 (StataCorp, College Station, TX) [23]. Inferences of statistically significant effects were based on a-priori defined significance level of alpha = 0.05 or if the 95% confidence interval overlapped the null value of incident rate ratio (IRR) = 1.00.

We then calculated the predicted number of institutional deliveries after 2012 using the fitted negative binomial model, under two scenarios: first, with months in their original rainy/dry season categorization; and second, with all months considered to be in the dry season. We summed the predicted counts for each scenario and took the difference as an estimate of how many women would not deliver at a facility because of the rain. We used the Lives Saved Tool (LiST) to model the increase in mortality due to this predicted decrease in utilization of institutional delivery associated with the rainy season. LiST is a mathematical modeling tool which allows users to model the impact of scaling up maternal, newborn, child health and nutrition (MNCH&N) interventions on mortality and nutritional outcomes [24]. Our predicted reduction in service utilization of institutional delivery corresponded to a 2% decrease. We therefore created a projection in LiST keeping utilization in 2020 at 64.8% and dropped it to 63.5% for 2021. We used 2020 for our base year and 2021 for our target year.

## Results

### Descriptive statistics

There was on average 515,622 women per month who completed ANC4 visits in the rainy season compared to 590,234 in the dry season. For institutional deliveries, the average for rainy season was 421,643 and 478,143 for dry season. Descriptive statistics are presented in Table 1.

**Table 1:** Number of observations, mean and ranges for rainfall, ANC4, and Institutional delivery, by province (2012–2015, 2017–2019)

	Rainfall (mm)			ANC4			Institutional Deliveries		
	n	Mean	Min-Max	n	Mean	Min-Max	n	Mean	Min-Max
Cabo-Delgado	86	85	3–385	73	6774	1659 - 11,578	73	5929	4287 - 8461
Gaza	86	50	8–345	73	5030	3043 - 6023	73	4086	1948 - 6391
Inhambane	86	65	9–352	73	4916	3138 - 6103	73	4043	2709 - 5469
Manica	86	75	9–463	72	7721	4668 - 12,174	73	6003	3993 - 8467
Maputo-Cidade	NA	NA	NA	73	3152	0–5043	73	3464	2296 - 5820
Maputo-Province	86	54	7–213	73	3821	2060 - 5583	72	3096	1957 - 5238
Nampula	87	86	5–376	73	21,989	8762 - 39,298	73	16,530	9072 - 23,601
Niassa	86	85	3–327	73	5916	2123 - 10,158	73	5878	3970 - 8283
Sofala	86	84	9–539	73	7839	4036 - 12,188	73	6635	4713 - 8858
Tete	86	69	2–360	73	8322	2912 - 14,128	73	6887	4358 - 9589
Zambezia	86	108	12–411	73	17,563	6861 - 33,929	73	12,994	8507 - 17,521

Abbreviations: *n* number of observations; *NA* Not applicable

**Fig. 1:**
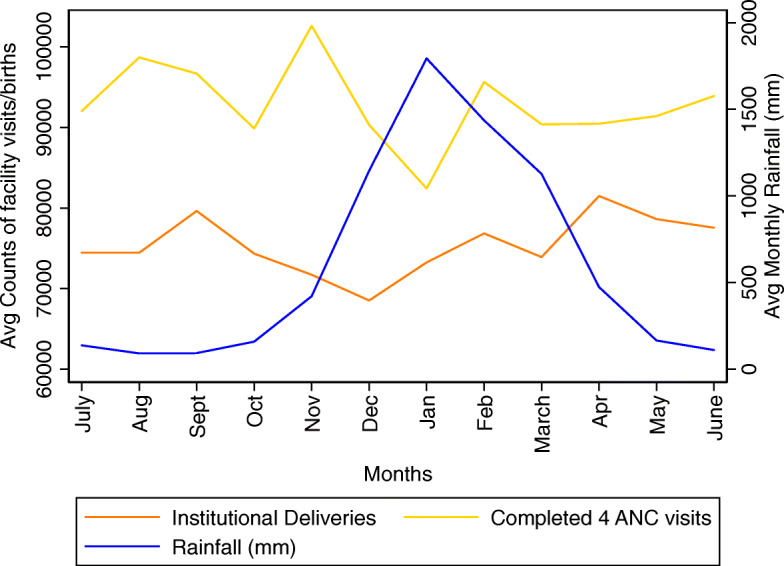
National pattern of ANC visits and Institutional Deliveries against precipitation data (2012–2015, 2017–2019)

Figure 1 shows the number of women completing four ANC visits each month and monthly deliveries at a health facility averaged per month over this time period nationally. Both of these indicate a decrease in frequency in the months with heavier rainfall (Fig. 1). Additional files show the monthly average for each year of data and by province [see Additional files 2, 3, 4 and 5].

### Negative binomial model analysis

Table 2 shows the results of the negative binomial model for the frequency of antenatal care utilization and institutional delivery in Mozambique. The rate of ANC4 is reduced by 1% for the rainy season compared to the dry season, adjusting for time and province (IRR = 0.99, 95% CI: 0.96–1.03). While not statistically significant, we still found a decrease in ANC4 during the

**Table 2:** Negative Binomial Regression Analysis Results for ANC4 and institutional delivery

	ANC4			Institutional delivery		
Variable	IRR	95% CI	***P*** value	IRR	95% CI	***P*** value
**Rainy Season**	0.99	0.96–1.03	0.606	0.94	0.92–0.96	< 0.001
**SISMA**	0.41	0.38–0.45	< 0.001	1.06	1.01–1.11	0.024
**Province**						
**Nampula (reference)**	1.00			1.00		
**Cabo-Delgado**	0.29	0.27–0.31	< 0.001	0.36	0.34–0.37	< 0.001
**Gaza**	0.26	0.24–0.28	< 0.001	0.24	0.23–0.26	< 0.001
**Inhambane**	0.24	0.22–0.26	< 0.001	0.24	0.23–0.25	< 0.001
**Manica**	0.37	0.34–0.40	< 0.001	0.36	0.34–0.38	< 0.001
**Maputo-Cidade**	0.15	0.14–0.16	< 0.001	0.22	0.21–0.23	< 0.001
**Maputo-Province**	0.18	0.17–0.20	< 0.001	0.19	0.18–0.20	< 0.001
**Niassa**	0.26	0.24–0.28	< 0.001	0.36	0.34–0.37	< 0.001
**Sofala**	0.36	0.33–0.39	< 0.001	0.40	0.39–0.42	< 0.001
**Tete**	0.38	0.35–0.41	< 0.001	0.41	0.39–0.43	< 0.001
**Zambezia**	0.78	0.72–0.84	< 0.001	0.78	0.75–0.82	< 0.001
**Time**	1.01	1.00–1.01	< 0.001	1.00	1.00–1.01	< 0.001

Abbreviations: *IRR* Incident Rate Ratio; *CI* Confidence Interval

rainy season.

For institutional deliveries, the rate of institutional deliveries is 6% lower during the rainy season than the dry season, after adjusting for time and province (IRR = 0.94, 95% CI: 0.92–0.96). This shows that the rainy season has a statistically significant lower rate of institutional deliveries compared to the dry season in Mozambique.

**Fig. 2:**
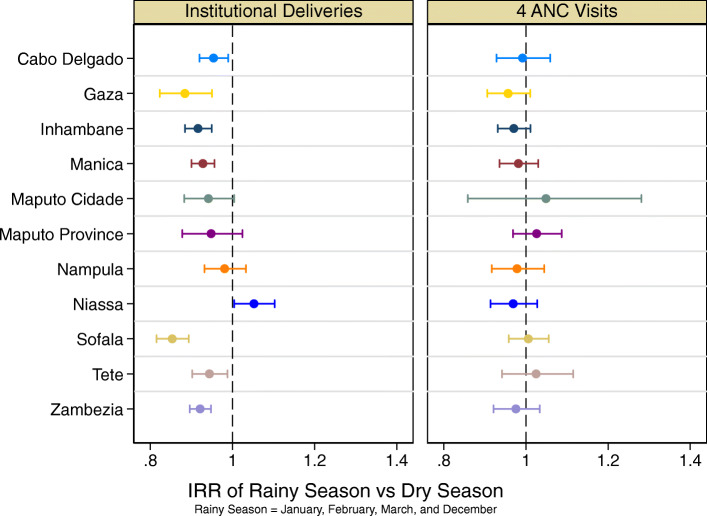
Comparison of model coefficients for the effect of rainy seasons on institutional delivery and ANC, by province

When analyzing institutional deliveries provincially, all provinces except for Maputo-Cidade, Maputo-Province, Nampula, and Niassa have a statistically significantly lower incident rate of institutional deliveries in the rainy season (Fig. 2, left). In contrast, provincially, there is no statistically significant difference between the rainy season and the dry season for ANC visits (Fig. 2, right).

### Quantifying the reduction in service delivery and excess mortality

**Fig. 3:**
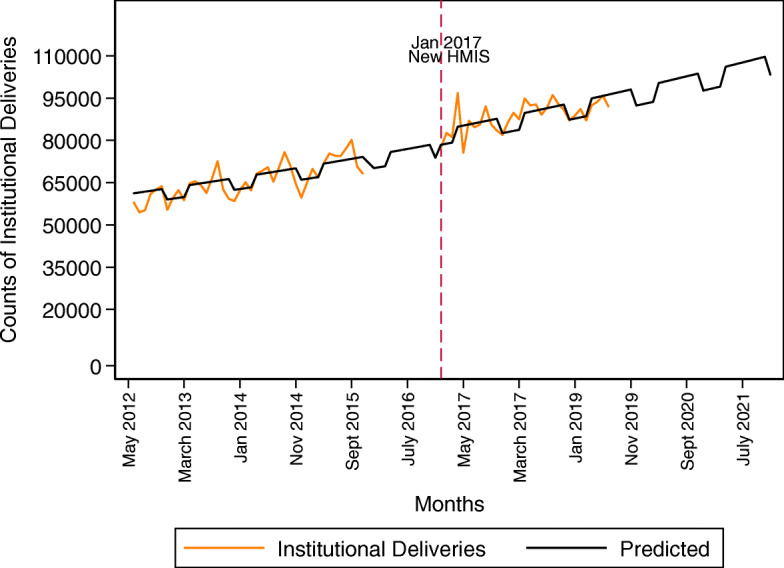
Predictive model (June 2012 – December 2019)

The predicted number of institutional deliveries in Mozambique in the original rainy/dry season categorization scenario and in the always dry season scenario are presented in Table 3. From our model, we can say that in 2021, approximately 26,546 women in Mozambique who would otherwise deliver in a facility will not do so during the rainy season. Figure 3 shows the predicted values superimposed onto the monthly count data of institutional delivery utilization at the national level.

**Table 3:** Predicted counts of institutional deliveries and difference due to the rainy season

Year^**a**^	Observed Count	Predicted count using model n (95% CI)	Predicted count using model if all months were non-rainy n (95% CI)	Difference due to rainy season
2013	755,837	762,995 (686,978 – 839,013)	779,040 (701,620 – 856,461)	16,045
2014	804,546	806,924 (726,530 – 887,318)	823,892 (742,014 – 905,770)	16,968
2015	793,959	798,851 (715,452 – 882,251)	813,170 (728,260 - 898,080)	14,319
2016	–	902,514 (812,596 – 992,431)	921,492 (829,915 - 1,013,069)	18,979
2017	1,014,220	1,009,141 (908,600 – 1,109,682)	1,030,362 (927,965 - 1,132,758)	21,221
2018	1,091,631	1,067,241 (960,911 – 1,173,570)	1,089,683 (981,391 - 1,197,975)	22,442
2019	–	1,128,686 (1,016,234 – 1,241,137)	1,152,420 (1,037,893 - 1,266,947)	23,734
2020	–	1,193,668 (1,074,742 – 1,312,594)	1,218,769 (1,097,649 - 1,339,890)	25,101
2021	–	1,262,392 (1,136,619 – 1,388,164)	1,288,938 (1,160,844 - 1,417,032)	26,546

^*a*^*Observed counts not included for years we do not have data for all 12 months*

Due to the rainy season in our model, we estimate that there would be 74 additional maternal deaths and 726 deaths of children under the age of 1 month (i.e. those most likely to be affected by institutional delivery outcomes) who would not otherwise die if the mother were able to deliver at a health facility from July 2020 to June 2021 (Table 4).

**Table 4:** Number of maternal and child deaths before and after decrease of institutional delivery utilization

	Assuming no effect of rainy season	With effect of rainy season	Difference	Percent increase
**Number of maternal deaths**	3297	3371	74	2.24%
**Number of < 1 month deaths**	31,704	32,430	726	2.29%

## Discussion

Our findings indicate that in Mozambique (1) season is significantly associated with institutional deliveries, whose counts are lower during the rainy season and that (2) while there is a decrease in counts for ANC4 during the rainy season, it is not statistically significant in most provinces.

These findings make sense given the nature of these two health interventions. While ANC visits should occur within given time periods (i.e. its recommended for the first contact to be at any time within the first trimester), there is no requirement for them to happen on a specific day. Therefore, if a woman intended to go to her ANC visit on a given day but the roads are inaccessible due to heavy rainfall, she can easily postpone her trip for another day when the rain has stopped and the roads are in better condition. She would still be within the recommended visit period window and therefore compliant with her ANC schedule. However, women have little say in when their labour starts, and correspondingly have no say in road or weather conditions on that day.

The link between high ANC4 rates and high institutional delivery attendance has been documented [25–28]. Given this, our findings suggest that the significant decrease in institutional delivery is likely not due to women choosing not to deliver in health facilities, but rather, not being physically capable of reaching the health facility in time. The implications of these findings are therefore two-fold: (1) that Mozambique has generated some interest for pregnant women to utilize maternal health services, and (2) that gains in maternal health are being missed, not because of the health sector but because of lack of investments in infrastructure and transportation options for pregnant women.

The governmental initiatives and strategic plans to improve maternal health indicate support from the government and desire to address obstacles to maternal health services [19]. Indeed, while women’s health (including maternal health) is being prioritized, gains in women’s health have been modest due to in part to poor implementation of government policies [29].

One solution to address the issue of access to maternal health services is to bring the services to the people who need them, either by using mobile clinics or by leveraging community health workers in remote areas. Mobile clinics are currently used in Mozambique to deliver primary health and maternal and child health services, along with HIV and TB services [30]. Community health workers are also responsible for providing pre-natal counseling and referrals to health facilities, but they do not provide ANC services themselves [31]. However, these options alone cannot close the gap. Firstly, the quality of maternal and child health services – particularly ANC4 – can vary significantly since it measures interaction with a skilled provider, but does not always speak to what service was received. Indeed, an analysis of 46 LMICs found that community-based ANC services had the lowest score for delivering all four ANC interventions [32]. Additionally, mobile clinics are often overwhelmed by demand, leading to rapid stock-outs of medication and patients not being able to be seen by a health care provider, which causes frustration and discourages returning [33].

Importantly, the mobile clinic approach is not suitable for institutional deliveries which by definition require access to a health facility. Studies in other settings have found that time to travel to delivery site [34] and availability of a clinic in the community [35] are associated with higher rates of institutional delivery. Maternity waiting homes are part of the national plan for health, but these too are imperfect solutions. Maternity waiting homes, if utilized, do have potential for improving maternal health outcomes. However, direct (e.g., transportation costs, food, lodging fees) and indirect (e.g., lost wages while at the waiting home, no available caretaker for other children) costs have been found to be significant barriers to the utilization of maternity waiting homes by a systematic review [36].

At a more upstream level, mother’s education is also associated with increased utilization of maternal health services [37], and access to education is also determined by roads and access to transport. Indeed, a 2015 study in rural Mozambique found lack of school and lack of access to health care services were most commonly cited as the biggest barrier to individuals’ and their families’ health [13].

Solutions to address problems related to access to health care must involve sectors outside the health sector. Gains on the health front are at risk of being lost unless infrastructure and transportations issues are addressed taking into account the meteorological realities of rural Mozambique. Every year, we estimate that 3297 mothers die due to reasons related to their pregnancy or childbirth. According to our analysis, around 2% of those deaths are related to the mere fact that mothers do not travel to a health facility because of the rain. While this is a small percentage, it shows how marginal factors relating to health system accessibility can have real-world, life-or-death consequences. A rural resident of Zambezia province described the problem succinctly: *“Access to health care is a big problem. I left my home this morning to reach here. We have a small clinic, but they don’t do blood tests. Sometimes we have transport, but today because of the rain it was difficult. It takes about 3 hours to walk, more if you are sick.”* [13].

Although our analysis estimated the number of maternal and child death that would not otherwise occur if the women delivered in the health facility but did not due to the rainy season, it is important to consider that there are other reasons why women do not deliver in health facilities. These may include maternal age, religion, parity, and exposure to information [38, 39].

### Limitations

Considering that only some facility-based data was available, a limitation is that we do not have demographic data on the women that are visiting the health facilities each month, such as their age, income level, or number of children*,* and we cannot account for potential inter- or intra-personal barriers to access to health services. Our analysis could have been strengthened if we had access to additional information about these women, such as distance from their residence to the health facility, to determine if they live in a rural region with limited road access, or far from the health facility. The generalization of the conclusions to all pregnant women in Mozambique is therefore limited. Peaks of health facility use in certain months that seem inconsistent with the overall trend could also be due to fertility patterns, occupational migration trends, or other patterns not captured in our data.

We also note that our conclusions are drawn from analyzing routine data. Routine data quality has been called into question with regards to completeness, timeliness, representativeness and accuracy. However, others have used routine data in Mozambique and argue that data in Mozambique’s HMIS platform was of sufficient quality to evaluate some health programs [40, 41], with one study reporting high availability and reliability of institutional delivery in particular. Of note, both studies refer to the pre-2016 *Modulo Basico* platform. We therefore caution that the interpretation of our results depends on the quality of the data contained in Mozambique’s HMIS platform.

There are other ways in which one can approach geo-spatial analysis. Several other groups have developed more sophisticated methods that involve geographical information system (GIS)-based spatial analysis, spatio-temporal modelling, or a combination of survey data and spatial analysis [15, 42, 43]. Others have considered uncertainty by incorporating spatial autocorrelation along with area overlap [44]. These types of analyses require specific types of data and complex methods. Since we were working with HMIS data and limited geographic data, we decided to limit the scope of our methods to a regression analysis of count data.

## Conclusion

In Mozambique, fewer women deliver at a health facility during the rainy season than during the dry season, meaning that fewer women have access to life-saving obstetric services when they need it. Until the role of infrastructure, specifically roads, is given consideration as a health systems priority, these geographical inequities will persist. This study highlights the need to address barriers to receiving care during pregnancy or childbirth. Due attention should be given to reducing geographic inequities, particularly in rural regions with a less developed road network. Doing so may require intersectoral collaboration, such as between the Ministry of Health and Ministry of Public Works and Housing – the department responsible for building and managing roads. While more research is needed to evaluate the impact of increasing extreme weather and heavy rainfall events on accessing health services, these results can help inform service delivery and infrastructure development programs.

## Supplementary information

**Additional file 1.** Precipitation Data, Nationally. Average monthly rainfall by year (PDF 65 KB)

**Additional file 2.** Institutional Deliveries by Month 2012–2019, Nationally. National monthly average of institutional deliveries for each year of data (PDF 60 KB)

**Additional file 3.** Completed ANC4 Visits by Month 2012–2019, Nationally. National monthly average of 4 completed ANC visits for each year of data (PDF 59 KB)

**Additional file 4.** Institutional Delivery Trends by Province (2012–2019). Average monthly rainfall and institutional deliveries by province (PDF 44 KB)

**Additional file 5.** ANC Trends by Province (2012–2019). Average monthly rainfall and 4 completed ANC visits by province (PDF 44 KB)

## Data Availability

The data that support the findings of this study are available from Mozambique’s Departamento de Planificação e Cooperação (DPC) but restrictions apply to the availability of these data, which were used under license for the current study, and so are not publicly available. Data are however available from the authors upon reasonable request and with permission of the DPC.

## Abbreviations

AIC: Akaike information criterion
ANC: Antenatal care
CHIRPS: Climate hazards group infrared precipitation with stations
CI: Confidence Interval
DHS: Demographic and health survey
HIV: Human immunodeficiency viruses
HMIS: Health management information system
IRR: Incident rate ratio
LiST: Lives saved tool
NA: Not applicable
PESS: Plano estratégico do sector da saúde
SIS-MA: *Sistema de informação para a saúde–monitoria e avaliação*
TB: Tuberculosis
TBA: Traditional birth attendant
USAID: United States Agency for International Development
VIF: Variance inflation factors
