# Supplementary material for: When it rains, it pours: detecting seasonal patterns in utilization of maternal healthcare in Mozambique using routine data
Source: BMC Health Serv Res. 2020 Oct 15;20:950. doi: 10.1186/s12913-020-05807-0 (PMC7559485; doi:10.1186/s12913-020-05807-0)
Supplement: Supplementary file 1 — Additional file 1. Precipitation Data, Nationally. Average monthly rainfall by year (PDF 65 KB) [file 12913_2020_5807_MOESM1_ESM.pdf]

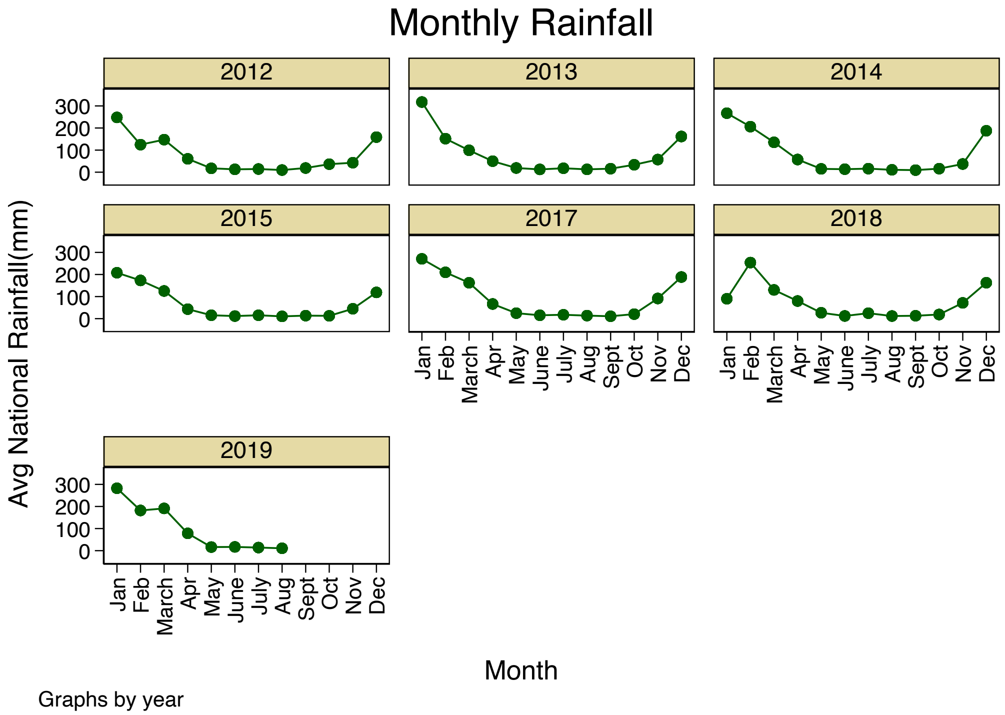
Monthly Rainfall
2012
2013
2014
2015
2017
2018
2019
300
200
100
0
Avg National Rainfall(mm)
Jan
Feb
March
Apr
May
June
July
Aug
Sept
Oct
Nov
Dec
Month
Graphs by year
