# Supplementary figures and images for: When it rains, it pours: detecting seasonal patterns in utilization of maternal healthcare in Mozambique using routine data
Source: BMC Health Serv Res. 2020 Oct 15;20:950. doi: 10.1186/s12913-020-05807-0 (PMC7559485; doi:10.1186/s12913-020-05807-0)

# Monthly Trends of Institutional Deliveries

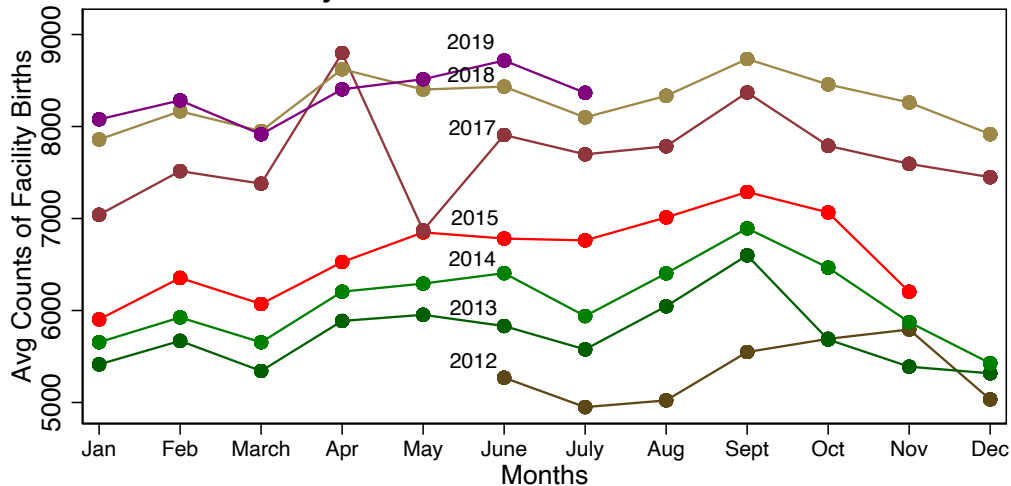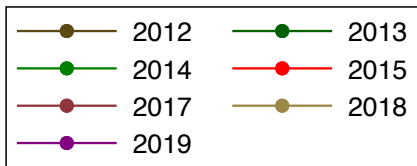

Supplement: Supplementary file 2 — Additional file 2. Institutional Deliveries by Month 2012–2019, Nationally. National monthly average of institutional deliveries for each year of data (PDF 60 KB) [file 12913_2020_5807_MOESM2_ESM.pdf]

# Monthly Trends of 4 Completed ANC Visits

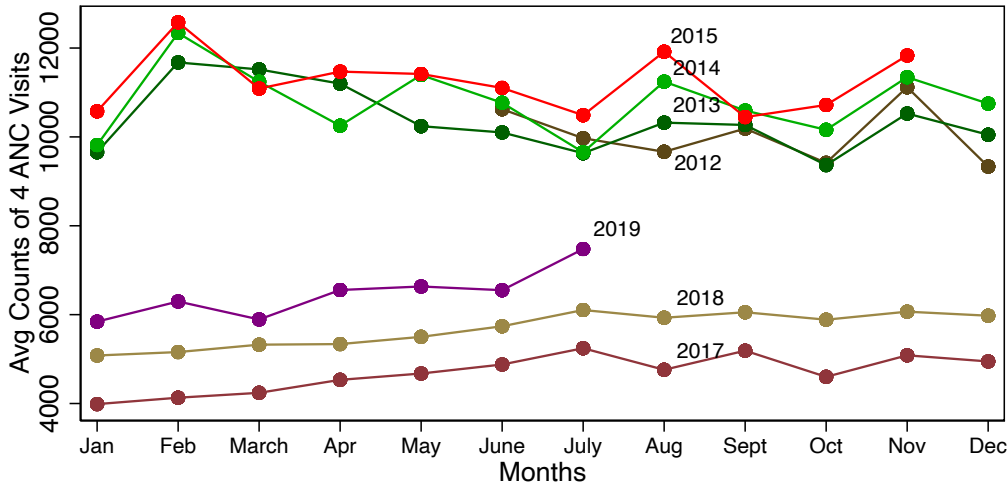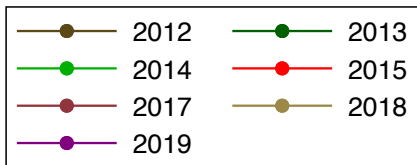

Supplement: Supplementary file 3 — Additional file 3. Completed ANC4 Visits by Month 2012–2019, Nationally. National monthly average of 4 completed ANC visits for each year of data (PDF 59 KB) [file 12913_2020_5807_MOESM3_ESM.pdf]

# Provincial Trends of Institutional Deliveries

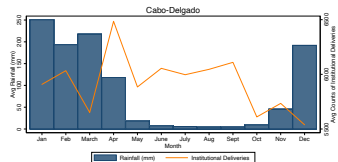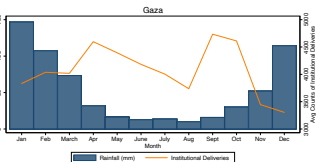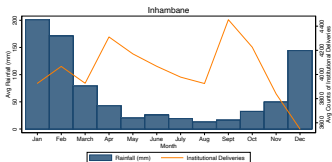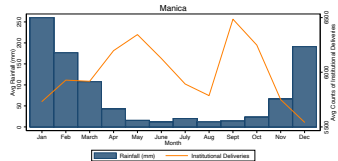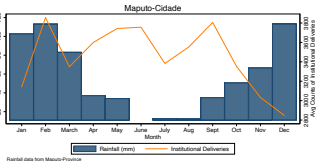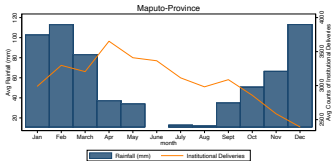

Rainfall data from Maputo-Province

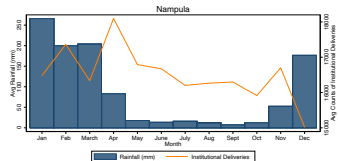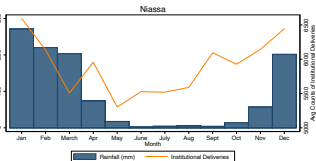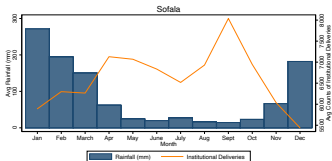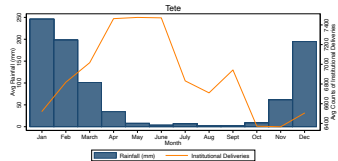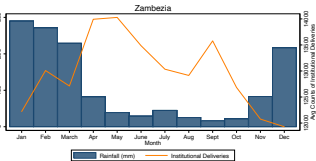

Supplement: Supplementary file 4 — Additional file 4. Institutional Delivery Trends by Province (2012–2019). Average monthly rainfall and institutional deliveries by province (PDF 44 KB) [file 12913_2020_5807_MOESM4_ESM.pdf]

# Provincial Trends of 4 Completed ANC Visits

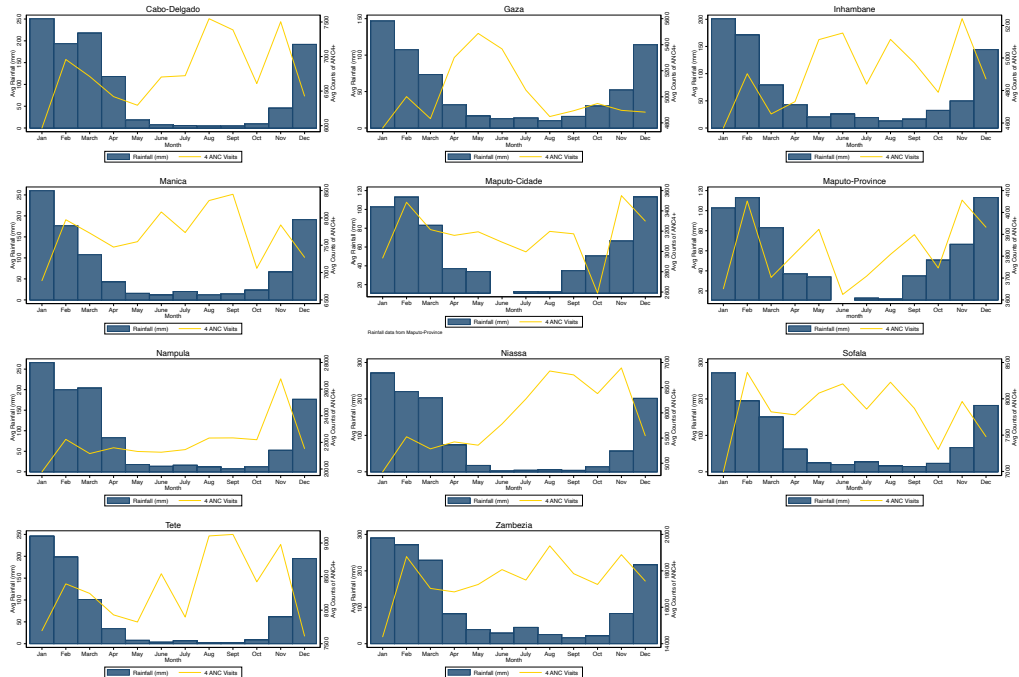

Supplement: Supplementary file 5 — Additional file 5. ANC Trends by Province (2012–2019). Average monthly rainfall and 4 completed ANC visits by province (PDF 44 KB) [file 12913_2020_5807_MOESM5_ESM.pdf]
